# Hippocampal sclerosis, TDP‐43, and the duration of the symptoms of dementia of AD patients

**DOI:** 10.1002/acn3.51135

**Published:** 2020-07-31

**Authors:** Oscar L. Lopez, Julia Kofler, YueFang Chang, Sarah B. Berman, James T. Becker, Robert A. Sweet, Neelesh Nadkarni, Riddhi Patira, M. Ilyas Kamboh, Ann D. Cohen, Beth E. Snitz, Lewis H. Kuller, William E. Klunk

**Affiliations:** ^1^ Department of Neurology University of Pittsburgh School of Medicine Pittsburgh Pennsylvania; ^2^ Department of Psychiatry University of Pittsburgh School of Medicine Pittsburgh Pennsylvania; ^3^ Department of Pathology University of Pittsburgh School of Medicine Pittsburgh Pennsylvania; ^4^ Department of Neurosurgery University of Pittsburgh School of Medicine Pittsburgh Pennsylvania; ^5^ Department of Psychology University of Pittsburgh School of Medicine Pittsburgh Pennsylvania; ^6^ Department of Medicine University of Pittsburgh School of Medicine Pittsburgh Pennsylvania; ^7^ Department of Human Genetics University of Pittsburgh School of Medicine Pittsburgh Pennsylvania; ^8^ Department of Epidemiology University of Pittsburgh School of Medicine Pittsburgh Pennsylvania

## Abstract

**Objectives:**

To examine the relationship between duration of the cognitive symptoms, from the earliest reported symptom to death, and hippocampal sclerosis (HS) and TAR‐DNA binding protein of 43kDA (TDP‐43) in Alzheimer’s disease (AD) patients.

**Methods:**

The study was conducted in 359 cognitively impaired patients who met the pathological criteria for AD (NIA‐Reagan intermediate or high). The mean age at onset was 69.5 ± 8.8 years (range 37‐95) and the mean duration of the symptoms was 10.5 ± 4.2 years. The association between symptoms duration and HS and TDP‐43 was examined with logistic regression analyses controlling for age at death, atherosclerosis in the Circle of Willis (CW), cerebral infarcts, gender, baseline Mini Mental State Examination scores, APOE‐4 allele, and presence of Lewy bodies (LB).

**Results:**

HS was present in 18% (n = 64) and TDP‐43 in 51.5% (n = 185) of the patients. HS and TDP‐43 were more frequent in patients whose symptoms lasted more than 10 years. LBs were present in 72% of the patients with HS and in 64% of the patients with TDP‐43. Age at onset was not associated with TDP‐43 or HS. HS was associated with duration of symptoms and LB, TDP‐43, and atherosclerosis in the CW. TDP‐43 was associated with duration of symptoms, LB, and HS.

**Interpretation:**

HS and TDP‐43 are present in early and late onset AD. However, their presence is mainly driven by the duration of symptoms and the presence of LB. This suggests that HS and TDP‐43 are part of the later neuropathological changes in AD.

## Introduction

The defining neuropathological features of Alzheimer’s disease (AD) are amyloid neuritic plaques and neurofibrillary tangles,^1^ but the majority of these patients have concomitant proteinopathies which are also common in other neurodegenerative processes.^2^ The transactive response DNA‐binding protein 43 (TDP‐43) is a neuropathological marker for frontotemporal lobar degeneration (FTLD) and amyotrophic lateral sclerosis,^3^ and is found in up to 57% of the patients with a neuropathological diagnosis of AD.^3^, ^4^, ^5^ In addition, TDP‐43 seems to be frequent in the oldest patients (age> 90),^4^ and it is also associated with the presence of Lewy bodies (LB) in individuals with and without dementia.^6^, ^7^ Recently, new terminology has been proposed for this TDP‐43 pattern, limbic‐predominant age‐related TDP‐43 encephalopathy (LATE), and the term LATE‐neuropathological change (NC) has been coined to describe a pathological entity that occurs in older adults with or without coexisting hippocampal sclerosis (HP) or AD pathology and is associated with a dementia syndrome similar to that seen in AD.^8^

HS is characterized by a disproportionate neuronal loss in the subiculum and CA1 regions of the hippocampus.^9^ HS is a common finding in community‐ ^10^ and population‐based cohorts,^11^, ^12^ its prevalence increases with age,^13^, ^14^, ^15^ and it is more frequent in women than men.^16^ HS can occur in isolation in a small number of elderly individuals,^12^ although it is more frequently associated with neurodegenerative processes and cerebrovascular disease.^9^ HS was reported present in up to 70% of the patients with FTLD with ubiquitin immunoreactive neuronal inclusions, and in up to 20% of the patients with pathologically diagnosed AD.^17^ Concurrent TDP‐43‐positive inclusions were found in 90% of the age‐associated HS cases.^13^

The purpose of this study is to examine the relationship among HS, TDP‐43, and LBs in pathologically diagnosed AD, and the duration of the cognitive symptoms. This has important implications for the understanding of the pathophysiological process. The relationship among the duration of symptoms, regardless the age of death, and these pathologies will indicate whether their presence is determined by the duration of the neurodegenerative process.

## Material and Methods

All of the subjects were participants in the University of Pittsburgh Alzheimer’s Disease Research Center (ADRC), and received an annual extensive neuropsychiatric evaluation including medical history and physical examination, neurological history and examination, semi‐structured psychiatric interview, and neuropsychological assessment.^18^, ^19^ At the end of these studies, each individual set of results was reviewed by a team of neurologists, neuropsychologists, and psychiatrists at a Consensus Conference. The inclusion and exclusion criteria have been published previously.^18^, ^19^ This study was approved by the local Institutional Review Board.

The ADRC has obtained 708 brains from 1985 to 2019 (604 with AD pathology and 104 without). From these, 421 had TDP‐43, HS, and LBs assessments at autopsy, and for this study, we selected 359 cases with pathologically confirmed AD as primary diagnosis, and who had progressive cognitive deficits, either mild cognitive impairment (MCI) or dementia during follow‐up. We excluded 12 individuals who were cognitively normal at the time of death, and 50 patients with Non‐AD pathologies. Of the 359 patients, 328 met criteria for clinical dementia at study entry, 20 for MCI (17 converted to dementia and 3 remained as MCI), and 11 were normal controls (10 converted to dementia, and 1 to MCI).

Age at onset was the age at which the earliest symptom was noted, and it was determined by independent interviews with the patients and proxies conducted by social workers, neurologists, and geriatric psychiatrists. In those patients who entered the study as normal controls, the age at onset was determined when they started reporting their first symptoms.

### Psychiatric examination

The psychiatric evaluations were conducted annually by geriatric psychiatrists using a semi‐structured interview and the Consortium to Establish a Registry for Alzheimer’s Disease (CERAD) Behavioral Rating Scale.^20^ In addition, the Hamilton Depression Rating Scale^21^ was completed by the geriatric psychiatrist based on data from each patient and primary caregiver. Following psychiatric assessments`, diagnoses of psychiatric syndromes were made according to the DSM‐IV.^22^

### Neurological examination

The neurological exam included a semi‐structured interview with the caregivers who enquired about the effects of cognition on the most relevant instrumental activities of daily living (IADLs) and performance in activities of daily living (ADLs) (e.g., household chores, driving, job performance, handling finances, hygiene, and sphincter control). The exam included cranial nerve testing, motor tone, abnormal movements, strength, deep tendon reflexes, release signs, plantar response and clonus, cerebellar testing, primary sensory testing (including graphesthesia and stereognosis), gait (including heel, toe, and tandem walking), and postural stability. The examiner also completed the CERAD Scale for parkinsonism^23^ and the Hachinski Ischemic Scale.^24^

### Neuropsychological exam

The neuropsychological evaluation has been modified over the 29 years spanning the clinical evaluation period, but consistently included global measures of cognition (i.e., the Mini‐Mental State Examination (MMSE)^25^ from 1983 to present, and the Montreal Cognitive Assessment (MOCA)^26^ from 2015 to present). Also consistently assessed were measures of episodic memory (e.g., CERAD,^27^ modified Rey‐Österreith figure^28^), Language (e.g., Modified Boston Naming Test^29^ and verbal fluency (FAS)^30^, ^31^), attention and executive functions (e.g., digit span,^32^ Trail making,^33^ and Stroop test), and Visuospatial and Visuoconstructional functions (e.g., Visual discrimination test,^34^ copy of the modified Rey‐Österreith,^28^ and modified Block Design^35^). Since 2005, the ADRC has also implemented the NACC‐UDS neuropsychological battery versions.^36^, ^37^

### MCI criteria

The MCI diagnosis was consistent with current diagnostic criteria.^38^ Patients with MCI amnestic type were individuals who had verbal and/or visual memory deficits with otherwise normal cognitive function. We also identified patients who had mild deterioration in one or more cognitive domains that could include memory, and patients with cognitive deficits in non‐memory domains, alone or in combination. These mild cognitive deficits may or may not have affected IADLs but did represent a decline from a previous level of functioning.^39^

### Diagnosis of dementia

The diagnosis of dementia was based on a deficit in performance in two or more cognitive domains that were sufficiently severe to affect the subjects’ instrumental activities of daily living, and history of normal intellectual function before the onset of cognitive abnormalities. The dementia criteria were designed to identify subjects with syndromes that could include relatively preserved memory functions, and thus a memory deficit was not required for the diagnosis of dementia. This clinical diagnosis of dementia has shown a 98% sensitivity and 88% specificity for AD.^19^

### Neuropathology

Brain banking procedures were approved by the Committee for Oversight of Research and Clinical Training Involving Descendants at the University of Pittsburgh. The brains were removed intact and weighed. The Circle of Willis (CW) was evaluated for distribution and severity of atherosclerotic lesions, anatomic variations, and aneurysms. Atherosclerotic lesions were graded as mild (≤30% stenosis), moderate (30–70% stenosis), and severe (≥70% stenosis) based on the most affected vessel. For the purpose of this study, the lesions were considered present only when they were graded severe or moderate. The forebrain was bisected mid‐sagittally, and the right hemisphere was further dissected fresh and frozen at −80°C. The left hemisphere was fixed in 10% formalin for 1‐2 weeks and sectioned in the coronal plane into 1 cm slabs. The slabs were inspected for any gross lesions, and then sampled for histological evaluation using standardized protocols. Given the extent of the brain collection period and retrospective nature of this study, pathology protocols that were regularly updated following published consensus guidelines. All brains were evaluated for neuritic plaque densities using modified Bielschowsky stains and classified as possible, probable, or definite AD following the CERAD criteria.^40^ The cases were also evaluated for the extent of neurofibrillary tangle pathology using Bielschowsky silver stain and/or tau immunohistochemistry to generate a Braak‐Braak stage,^41^, ^42^ and National Institute on Aging and Reagan Institute (NIA‐RI) category of low, intermediate, or high likelihood that dementia was due to AD lesions.^43^, ^44^ The brains were classified in the lower NIA‐RI category if there was a discordance between the Braak‐Braak stage and the CERAD plaque score. Information about Aβ‐amyloid Thal phases and NIA‐Alzheimer’s Association ABC score was only available for a subset of cases,^45^ and consequently was not included in this study. In this study, we included all cases with NIA‐RI category of high (n = 278) and intermediate (n = 81) likelihood of AD.

LB pathology was assessed by alpha‐synuclein immunohistochemistry (LB509, 1:300, protease XXIV pretreatment; Invitrogen, Thermo Fisher, Waltham, MA), and positive cases were classified into amygdala, brainstem, limbic, neocortical, or other location subtypes. Older cases were restaged using the updated NIA‐AA consensus criteria^1^ and DLB consortium.^46^ For this study, the presence of LB was dichotomized as present or absent.

Immunohistochemical staining for phospho‐TDP‐43 was performed on sections of amygdala, hippocampus‐mesial temporal cortex, and midfrontal and inferior temporal neocortical regions (1D3, 1:500, citrate; kindly provided by Manuela Neumann, Helmholtz Zentrum, Munich, Germany, or purchased from Millipore Sigma, Burlington, MA).^47^ Sections were evaluated for the presence or absence of TDP‐43‐positive neuronal cytoplasmic inclusions, neuronal intranuclear inclusions, and dystrophic neurites. Based on the distribution of TDP‐43 pathology, positive cases were classified into amygdala predominant (present in amygdala only), mesial temporal (present in hippocampus, subiculum, and/or mesial temporal lobe in addition to the amygdala), or neocortical (present in midfrontal cortex and/or inferior temporal lobe in addition to the mesial temporal lobe structures).^5^ A case was considered positive for TDP‐43 if any abnormal inclusions were present in at least one of the analyzed brain regions. In the dichotomized analyses, all three TDP‐43 pathology stages were included in the TDP‐43‐positive group.

HS was evaluated in a coronal section of the anterior hippocampus and a second section of the mid‐hippocampus at the level of the lateral geniculate body. It was considered present based on severe neuronal loss and gliosis in CA1 and/or subiculum that was out of proportion to AD pathology in the same regions.^1^

All cases were reviewed by a single experienced neuropathologist (JK) to confirm original interpretations and to assure uniform scoring. All immunohistochemical studies included appropriate positive and negative control slides to exclude technical errors.

### Statistical analysis

The data were analyzed using chi‐square, t‐test, and ANOVA methods. The association among HS, LB, and TDP‐43 was examined with binary logistic regression models that controlled for gender, MMSE score, apolipoprotein E‐4 (APOE*4) allele, age at death, duration of the symptoms, lacunes and micro‐ and macroinfarcts, atherosclerosis in the Circle of Willis, and presence of LB. The analyses were conducted with age at death and duration of the symptoms as continuous variables.

## Results

The mean age at onset was 69.5 ± 8.7 and the mean duration of the symptoms was 10.5 ± 4.1. HS was present in 17.8% (n = 64) patients and TDP‐43 in 51.5% (n = 185). Table 1 shows the demographic, clinical, and pathological features of the AD patients with and without HS. Patients with HS were older at death, more likely to died after age 80, to be women, had a longer duration of the symptoms, had more moderate or severe atherosclerosis in the CW, and had more frequent LB and TDP‐43 than those without HS. There was a greater proportion of HS patients with TDP‐43 in the mesial temporal lobe and in neocortical regions than those without. HS patients had more micro‐ and macroinfarcts than those without HS (Cramer’s V = 20), but the difference was not statistically significant (*P* = 0.07). Table 2 shows the demographic, clinical, and pathological features of the AD patients with and without TDP‐43. AD patients with TDP‐43 were older at death, more likely to die after age 80, had a longer duration of the symptoms, had more LB and HS, and had more atherosclerosis in the CW than those without TDP‐43.

**Table 1:** Demographic and pathological characteristics of AD patients with hippocampal sclerosis (n = 359).

	Hippocampal sclerosis		Effect size	*P*‐value
	Present (%)	Absent (%)		
Number of patients	64 (18)	295(82)		
Age at onset	69.7 ± 8.3	69.4 ± 8.7	0.00	0.80
<age 75	46 (72)	206 (70)	0.017	0.74
>age 75	18 (28)	89 (30)		
Age at study entry	74.5 ± 7.7	73.2 ± 8.7	0.004	0.23
Age at death	83.4 ± 8.0	79.3 ± 8.5	0.035	<0.001
<80 years	22 (34)	157 (53)	0.144	0.006
>80 years	42 (66)	138 (47)		
Follow‐up time, in years	13.2 ± 4.6	9.8 ± 3.7	0.105	<0.001
<10 years	13 (23)	153 (52)	0.213	<0.001
>10 years	49 (77)	142(48)		
Gender (Women)	47 (73)	157 (53)	0.156	0.003
Education level	13.0 ± 3.1	13.5 ± 3.3	0.003	0.29
APOE‐4 allele (n = 357)	46 (69)	166(59)	0.09	0.06
Baseline Cognitive measures				
MMSE at baseline	18.4 ± 6.3	18.4 ± 6.3	0.000	0.97
CDR	1.26 ± 0.75	1.26 ± 0.65	−0.000	0.99
Baseline cerebrovascular risk factors				
Hypertension	28 (44)	115 (39)	0.037	0.48
Heart disease	13 (20)	89 (30)	0.084	0.11
Diabetes Mellitus	4 (6)	17 (6)	0.008	0.88
Other autopsy findings				
Lewy bodies	46 (72)	159 (54)	0.139	0.008
TDP‐43	52 (81)	133(45)	0.277	<0.001
Amygdala TDP‐3	10 (16)	50 (17)	0.359	<0.001
Mesial temporal lobe TDP‐43	22 (34)	63 (21)		
Neocortical TDP‐43	20 (31)	20 (7)		
NIA‐Reagan				
High	50 (81)	216 (75)	0.060	0.25
Intermediate	12 (19)	72 (25)		
CERAD				
Definite	63 (98)	286 (97)	0.035	0.51
Probable	1 (2)	9 (3)		
Braak & Braak Stage				
3	2 (3)	22 (7.5)	0.108	0.24
4	8 (12.5)	48 (16)		
5	12 (19)	70 (24)		
6	42 (66)	155 (52.5)		
Moderate or severe atherosclerosis in the CW	44 (69)	132 (45)	0.184	<0.001
Lacunes, and micro‐ and large infarcts	29 (45)	99 (34)	0.094	0.07

MMSE: Mini Mental State Examination; CDR: Clinical Dementia Rating, obtained at the time of study entry in dementia and MCI patients and at the time of diagnosis change in normal patients who converted to MCI.

NIA‐RI: National Institute on Aging – Reagan Institute; CERAD: Consortium to Establish a Registry for Alzheimer's Disease. CW: Circle of Willis.

Effect size: Cramer’s V for categorical data and Cohen’s f^2^ for continuous data.

**Table 2:** Demographic and pathological characteristics of AD patients with TDP‐43 (n = 359).

	TDP‐43		Effect size	*P*‐value
	Present (%)	Absent (%)		
Number of patients	185 (51.5)	174 (48.5)		
Age at onset	69.8 ± 8.8	69.8 ± 9.3	0.02	0.43
<75 years	131 (71)	121 (69.5)	0.014	0.79
>75 years	54 (29)	53 (30.5)		
Age at study entry	74.2 ± 8.0	72.7 ± 8.9	0.008	0.10
Age at death	81.4 ± 8.0	78.5 ± 8.8	0.030	0.001
<80 years	82 (44)	97 (56)	0.114	0.03
>80 years	103 (56)	77 (44)		
Follow‐up time, in years	11.4 ± 4.4	9.4 ± 3.5	0.058	<0.001
<10 years	69 (37)	99 (57)	0.196	<0.001
>10 years	116 (63)	75 (43)		
Gender (Women)	111 (60)	93 (53)	0.066	0.21
Education level	13.2 ± 3.3	13.7 ± 3.3	0.004	0.21
APOE‐4 allele	116(63)	104 (60)	0.030	0.57
Baseline Cognitive measures				
MMSE at baseline	18.1 ± 6.0	18.6 ± 6.3	0.002	0.46
CDR	1.24 ± 0.61	1.28 ± 0.72	0.001	0.58
Baseline cerebrovascular risk factors				
Hypertension	71 (38)	72 (41)	0.031	0.56
Heart disease	50 (27)	52 (30)	0.032	0.54
Diabetes Mellitus	11 (6)	10 (6)	0.004	0.93
Other autopsy findings				
Lewy bodies	118 (64)	87 (50)	0.319	0.008
Hippocampal sclerosis	52 (28)	12 (7)	0.277	<0.001
NIA‐RI				
High	145 (78)	133 (76)	0.023	0.66
Intermediate	40 (22)	41 (24)		
CERAD				
Definite	181 998)	168 (97)	0.039	0.45
Probable	4 (2)	6 (3)		
Braak & Braak Stage				
3	10 (5)	14 (8)	0.081	0.50
4	28 (15)	28 (16)		
5	39 (21)	43 (25)		
6	108 (58)	89 (51)		
Moderate or severe atherosclerosis in the CW	103 (56)	73 (42)	0.137	0.009
Lacunes, and micro‐ and large infarcts	66 (36)	53 (30.5)	0.024	0.65

MMSE: Mini Mental State Examination; CDR: Clinical Dementia Rating, obtained at the time of study entry in dementia and MCI patients and at the time of diagnosis change in normal patients who converted to MCI. CW: Circle of Willis.

NIA‐RI: National Institute on Aging – Reagan Institute; CERAD: Consortium to Establish a Registry for Alzheimer's Disease

Effect size: Cramer’s V for categorical data and Cohen’s f^2^ for continuous data.

The proportion of AD patients with HS alone (n = 12), TDP‐43 alone (n = 133), or both (n = 52) is shown in Table 3. AD patients with both HS and TDP‐43 were older at the time of death and had longer duration of the symptoms compared to those without any lesion. Patients with HS, TDP‐43, or both were more likely to have LB, those with HS alone or in combination with TDP‐43 had moder moderate and severe atherosclerosis in the CW than those without any lesion. The MMSE score was lower in those with HS compared to the other groups.

**Table 3:** Demographic and pathological characteristics of the AD patients with hippocampal sclerosis and TDP‐43 (n = 359).

	None (%)	Hippocampal sclerosis (%)	TDP‐43 (%)	Both (%)	Effect size	*P*‐value
Number of patients	162 (45.1)	12 (3)	133 (37)	52 (14.5)		
Age at onset	69.3 ± 9.2	65.8 ± 11.1	69.5 ± 8.2	70.6 + 7.4	0.009	0.36
<75 years	112 (69)	9 (75)	94 (71)	37 (71)	0.027	0.96
>75 years	50 (31)	3 (25)	39(29)	15 (29)		
Age at study entry	72.9 ± 9.0	70.8 ± 8.4	73.7 ± 8.3	75.5 ± 7.3	0.014	0.16
Age at death	78.6 ± 8.9	77.4 ± 8.6	80.1 ± 7.9	84.8 ± 7.2	0.065	<0.001
<80 years	90 (56)	7 (58)	67 (50)	15 (29)	0.180	0.009
>80 years	72 (44)	5 (42)	66 (50)	37 (71)		
Follow‐up time, years	9.3 ± 3.5	11.6 ± 3.4	10.5 ± 3.9	13.5 ± 4.8	0.136	<0.001
<10 years	95 (59)	4 (33)	58 (44)	11 (21)	0.257	<0.001
>10 years	67 (41)	8 (67)	75 (56)	41 (79)		
Women	82 (51)	11 (92)	75 (56)	36 (69)	0.181	0.008
Education level	13.7 ± 3.3	12.7 ± 2.4	13.3 ± 3.3	13.1 ± 3.2	0.008	0.44
APOE‐4 allele (n = 357)	93(58)	11 (92)	81 (61)	35 (67)	0.133	0.09
Baseline Cognitive measures						
MMSE at baseline	18.9 ± 6.1	14.5 ± 8.3	17.7 ± 6.2	19.3 ± 5.2	0.024	0.03
CDR	1.26 ± 0.70	1.62 ± 0.93	1.27 ± 0.59	1.18 ± 0.68	0.012	0.23
Baseline cerebrovascular risk factors						
Hypertension	68 (42)	4 (33)	47 (35)	24 (46)	0.084	0.47
Heart disease	51 (31.5)	1 (8)	38 (29)	12 (23)	0.104	0.27
Diabetes mellitus	10 (6)	0 (0)	7 (5)	4 (8)	0.057	0.75
Other autopsy findings						
Lewy bodies	78 (48)	9 (75)	81 (61)	37 (71)	0.182	0.008
NIA‐RI						
High	112 (75)	11(92)	103 (77)	43 (81)	0.077	0.54
Intermediate	40 (25)	1 (8)	30 (23)	10 (19)		
CERAD						
Definite	105 (65)	9 (75)	91 968)	26 (50)	0.056	0.77
Probable	57 (35)	3 (25)	42 932)	26 (50)		
Braak & Braak Stage						
3	13 (8)	1 (8)	9 (7)	1 (2)	0.083	0.58
4	28 (17)	0 (0)	20 (15)	8 (14)		
5	40 (25)	3 (25)	30 (23)	9 (17)		
6	81 (50)	8 (67)	74 (56)	34 (65)		
Moderate or severe atherosclerosis in the CW	65 (40)	8 (67)	67 (50)	36 (69)	0.206	0.002
Lacunes, and micro‐ and large infarcts	57 (35)	3 (25)	42 (32)	26 (50)	0.132	0.10

MMSE: Mini Mental State Examination; CDR: Clinical Dementia Rating. NIA‐RI: National Institute on Aging – Reagan Institute; CERAD: Consortium to Establish a Registry for Alzheimer's Disease. CW: Circle of Willis

Effect size: Cramer’s V for categorical data and Cohen’s f^2^ for continuous data.

Table 4 shows the proportion of patients with TDP‐43 and HS stratified by duration of the symptoms (A), age at death (B), and by age at onset (C). Both TDP‐43 and HS were most common among the patients followed for >15 years. TDP‐43 was most common in patients who died after age 75, and HS in those who died after age 80. No statistical differences were noted by age at onset of the symptoms.

**Table 4:** TDP‐43 and hippocampal sclerosis stratified by years of duration of the symptoms from first reported symptom to death, by age at death, and by age at onset of the symptoms (n = 359).

Follow up in years	Number of patients	TDP‐43 present (%)	Hippocampal sclerosis present (%)
A: By years of duration of symptoms			
0–5	20	6 (30)	1 (5)
5–9	147	62 (42)	14 (9.5)
10–15	149	85 (57)	30 (20)
>15	43	32 (74)	19 (44)
		X^2^ = 19.6, *P* = <0.001	X^2^ = 30.1, *P* = <0.001
B: By age at death			
<70	48	16 (33)	2 (4)
70–74	40	17 (42.5)	4 (10)
75–79	80	41 (51)	14 (17.5)
80–84	85	43 (51)	17 (20)
85–90	68	45 (66)	15 (22)
>90	38	23 (60.5)	12 (32)
		X^2^ = 14.7, *P* = 0.01	X^2^ = 13.8, *P* = 0.01
C: By age at onset of the symptoms			
<60	41	17 (41.5)	5 (12)
60–64	57	31 (54)	11 (19)
65–69	64	33 (52)	13 (20)
70–74	91	51 (56)	17 (19)
74–79	66	33 (50)	9 (14)
>80	40	20 (50)	9 (22.5)
		X^2^ = 2.69, *P* = 0.74	X^2^ = 2.67, *P* = 0.75

Table 5 shows the fully adjusted logistic regression model results. In the fully adjusted model, the presence of HS was associated with duration of the symptoms, moderate or severe atherosclerosis in the CW, LB, and TDP‐43. TDP‐43 was associated with duration of the symptoms, LB, and HS. The analyses were similar when we entered dichotomized measures of duration of the symptoms (<10/>10 years, follow up median was 10 years) and age at death (<80/>80, age at death median was 80 years).

**Table 5:** Factors associated with TDP‐43 and Hippocampal sclerosis in patients with pathological AD using age and duration of symptoms as dichotomized variables.

All Predictors in the Model	Hippocampal sclerosis		TDP‐43		
	OR (95% CI)	*P*‐value	OR (95% CI)	*P*‐value
Age at death	1.04 (0.99–1.09)	0.09	1.03 (1.00–1.06)	0.051
Duration of the symptoms	1.16 (1.07–1.25)	0.0003	1.09 (1.02–1.16)	0.009
Lewy bodies	2.76 (1.35–5.63)	0.005	1.70 (1.06–2.72)	0.02
TDP‐43	3.34 (1.63–6.83)	0.001		
Hippocampal sclerosis			3.43 (1.69–6.97)	0.001
Moderate or severe atherosclerosis in the Circle of Willis	1.99 (1.02–3.89)	0.04	1.25 (0.77–2.05)	0.36
Lacunes, and micro‐ and large infarcts	1.55 (0.80–3.02)	0.19	0.94 (0.57–1.54)	0.79
Gender	0.59 (0.30–1.16)	0.12	1.05 (0.66–1.67)	0.82
MMSE at study entry	0.99 (0.93–1.04)	0.57	0.98 (0.94–1.01)	0.20
APOE‐4 allele	1.91 (0.96–3.82)	0.06	1.01 (0.63–1.60)	0.98

**Figure 1:**
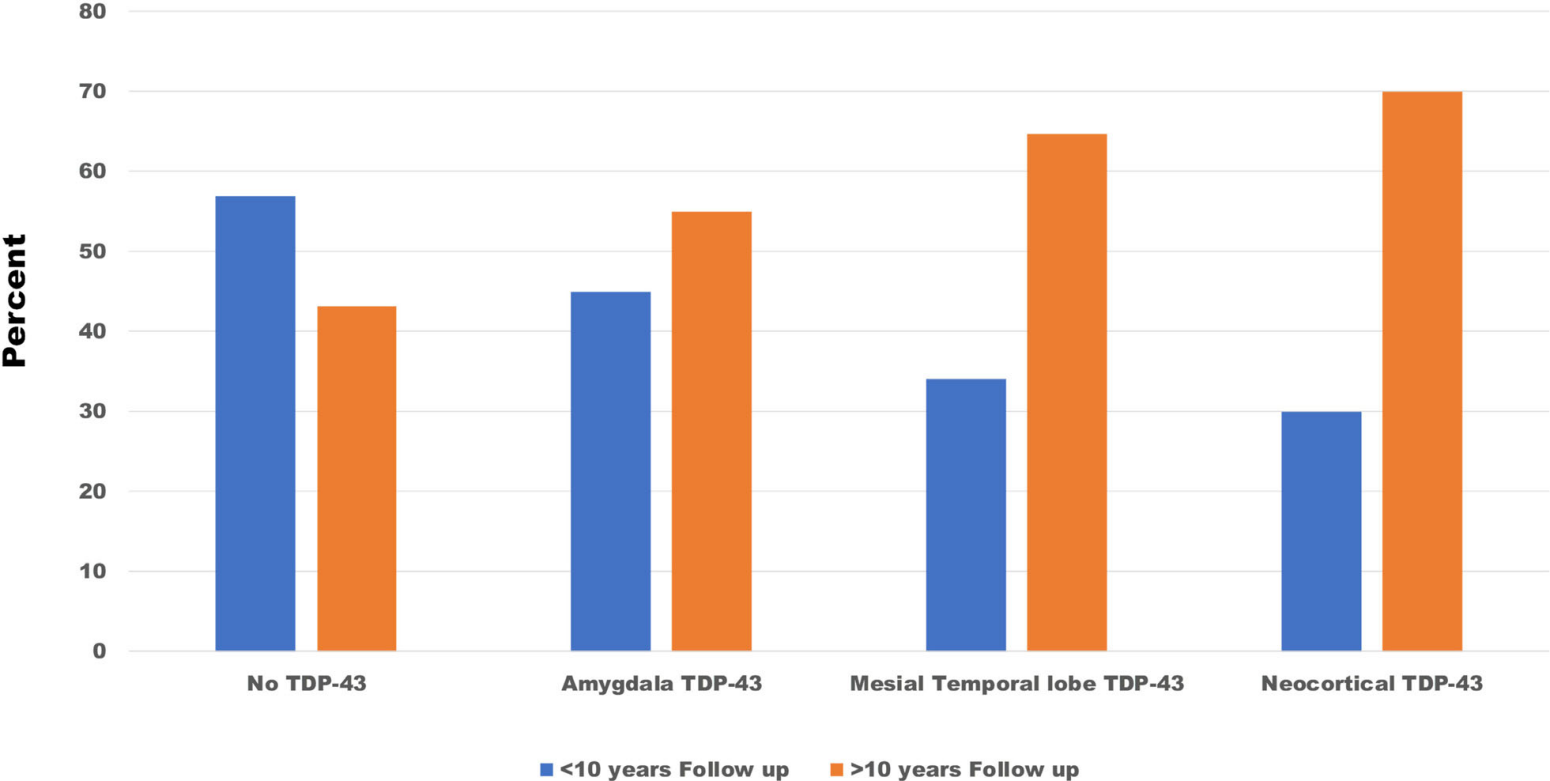
Regional TDP‐43 distribution by years of follow up.

Of the 185 patients with TDP‐43, 60 had TDP‐43 in the amygdala, 85 in the mesial temporal lobe, and 40 in neocortical regions (see also Table 1). Table 6 shows the relationship between the TDP‐43 stages and HS. Of the stages examined, mesial temporal and neocortical TDP‐43 were associated with HS, but not amygdala TDP‐43, and the association between HS and duration of the symptoms and LB remained statistically significant. Figure 1 shows the distribution of TDP‐43 in the different brain regions by duration of the symptoms dichotomized as <10/>10 years. There were more patients with >10 years of follow up with TDP‐43 in the mesial temporal lobe and neocortical regions than those with <10 years of follow up (χ^2^ = 16.2, p = 0.001).

**Table 6:** Relationship between TDP‐43 stages and hippocampal sclerosis.

	HR (95% CI)	*P*‐value
Age at death	1.04 (0.99–1.09)	0.09
Duration of symptoms	1.17 (1.08–1.27)	<0.0001
Lewy bodies	2.71 (1.31–5.63)	0.007
TDP‐43 stages		
Amygdala	1.60 (0.59–4.32)	0.35
Mesial temporal	2.75 (1.21–6.27)	0.01
Neocortical	10.83 (4.15–28.26	<0.0001
Moderate or severe atherosclerosis in the Circle of Willis	1.92 (0.96–3.83)	0.06
Lacunes, and micro‐ and large infarcts	1.84 (0.92–3.70)	0.08
Gender	0.63 (0.32–1.25)	0.18
MMSE	0.98 (0.93–1.04)	0.50
APOE‐4 allele	2.09 (1.01–4.32)	0.04

## Discussion

Our findings are consistent with previous observations of an association between HS, TDP‐43, and old age in demented patients. The proportion of cognitively impaired individuals with both pathologies increased with age at death in AD patients. However, the critical factor is the duration of the symptoms not older age *per se* (i.e., Table 5). This suggests that HS and TDP‐43 are part of the gradually progressive nature of the neurodegenerative process and are a late‐stage phenomenon of AD pathology. It is important to point out that we used a crude measurement of estimates of the duration of the dementia. Ideally, it would have been measured from the time that *in vivo* biomarkers become positive until death. Future cohorts with large number of individuals with *in vivo* biomarkers and autopsy will be able to better understand this relationship. We have also examined the relationship between duration of the symptoms from the time of study entry to death (mean follow up 6.01 years); TDP‐43 (OR: 1.12 (1.04–1.20, *P* = 0.004) and HS (OR:1.17 (1.07–1.29, *P* = 0.001) were associated with duration of the symptoms.

Why the core AD pathology (Aβ and tau) coexists with other markers of neurodegeneration, such as TDP‐43, HS, or LB is unknown. However, our findings suggest that various markers of neurodegeneration can influence each other if given enough time to interact, although the factors that trigger this process are unknown. There may be a synergistic effect among α‐synuclein, tau, and Aβ amyloid proteins,^48^ which could be determined by genetic factors.^49^ In animal models, the levels of Aβ, possibly in a permissive environment, can promote formation of α‐synuclein and tau filaments.^50^ These mixed pathologies may also be explained by activation of the microglia by Aβ deposition, which subsequently affects neurons and lead to other markers of neurodegeneration.^2^ Importantly, the association between HS and TDP‐43 and the duration of the symptoms occurred only in AD. Although not shown here, we have conducted similar analyses in patients without pathological AD, mainly FTD, and we could not find any association, suggesting that TDP‐43 and HS in these other diseases are part of the leading pathological process.

The relationship between HS and duration of the symptoms seems to be stronger in those individuals with AD pathology who died after age >80. Whether HS is an independent process that occurs in parallel to AD or is caused by AD cannot be determined in this study. The most likely explanation is that those who live many years with the disease are at risk of HS, as a late manifestation of AD neurodegeneration. Our results are consistent with previous reports on the increased prevalence of HS in the oldest (age >90), and an association among HS, TDP‐43, and LB.^13^ By contrast, other studies did not find an association between HS and LB in individuals with a mean age at death >88.^4^ There were more women than men with HS consistent with previous findings,^16^ possibly reflecting the increased prevalence of AD in women. However, the association was attenuated in the multivariate analysis.

We did not examine the clinical symptoms associated with these pathological markers. However, the AD cases with only HS had lower MMSE scores at baseline. This can be explained by the fact that four of these patients had MMSE scores < 10 at study entry and had symptoms of dementia for 6–14 years before the initial visit. Previous studies have found that patients with HS have lower MMSE scores, more memory problems than those without,^4^, ^13^ and those with TDP‐43 have more hippocampal atrophy in MRI scans during life.^51^

Studies conducted in >90 years old find a weak association between HS and cerebral infarcts.^13^ Although the proportion of patients with micro‐ and macroinfarcts was higher in the HS group, alone, or in combination with TDP‐43, when compared to those without HS, we could not find an association between infarcts and HS and TDP‐43 in the multivariate model. The factors associated with micro‐ and macroinfarcts were older age at onset of the symptoms (OR: 1.07, 95% CI: 1.04–1.10, *P* < 0.001) and duration of the symptoms (OR: 1.06, 95% CI: 1.00–1.13, *P* = 0.03). Therefore, it is possible that this association exists, but it may be circumscribed to old–old individuals.

We found an association between moderate‐severe atherosclerosis in the CW and HS, which reflects a life‐time evolution of arterial deposition of fatty acids that leads to atheromatous plaques. Indeed, atherosclerosis in the CWs was associated with age at dead (OR: 1.10, 95% CI: 1.06–1.14, *P* < 0.001) and HS (1.99, 95% CI: 1.05–3.80, *P* = 0.03). There are studies that found an association between TDP‐43 and arteriolosclerosis, which is also a consequence of a long‐standing vascular process, but not with atherosclerosis.^52^ In this cohort, the proportion of individuals with atherosclerosis and HS increased with age; atherosclerosis in the CW went from 10% in those who died before age 70 to 74% of those who died after age 90, and 75% of those who died after age 90 with HS had atherosclerosis in the CW.

Our findings are consistent with the notion that TDP‐43 pathology has a hierarchical distribution, which starts in the amygdala and progresses over time to neocortical regions.^8^, ^53^, ^54^ As noted in Figure 1, there was a greater proportion of patients with more than 10 years follow up with mesial temporal and neocortical TDP‐43. This hierarchical distribution may also determine the association with HS. Patients with HS had a greater proportion of mesial temporal and neocortical TDP‐43, but not amygdala TDP‐43. The multivariate analysis confirmed this association, and the point estimates of the association with HS increased with a greater regional distribution of TDP‐43 from the amygdala to mesial temporal to neocortical.

It is possible that patients with TDP‐43 and HS had longer disease duration of the symptoms because their symptoms started with lower AD pathology. This is difficult to test in neuropathological series because of the lack of *in vivo* biomarkers for these two pathologies. However, there were no statistical differences in the Braak & Braak staging among the cases with TDP‐43, HS, or both, and consequently, it is less likely that TDP‐43 and HS started with lower AD pathology.

One limitation of our database is that we did not examine individuals who died without cognitive impairments, which precluded an analysis of the presymptomatic disease. However, the wide age range at symptoms onset and age at death allowed us to examine the relationship between these variables and HS and TDP‐43 across different age stages, including individuals who are not usually recruited in longitudinal observational studies of the elderly people. We have shown that HS and TDP‐43 are present in individuals who developed AD before age 60 and died before age 70. Indeed, 9% (17/185) of the AD patients with TDP‐43 and 8% (5/64) of those with HS developed dementia before age 60. In addition, we have noted that these pathological lesions are also present in patients with a short disease duration. Additional studies are necessary to explore the clinical and pathological features of these individuals.

## Author Contributions

Conception and design of the study: OLL, JK, JTB, and YFC. Acquisition and analysis of data: OLL, JK, YFC, JTB, RS, LHK, SB, AC, IMK, BS, NN, RP, and WEK. Drafting a significant portion of the manuscript or figures: OLL, JK, YFC, JTB, LHK, SB, IMK, and WEK,

## Conflicts of Interest

None of the authors has any real or apparent conflict of interest or any commercial relationships with the material presented in the study.
